# Semantic Similarity in Biomedical Ontologies

**DOI:** 10.1371/journal.pcbi.1000443

**Published:** 2009-07-31

**Authors:** Catia Pesquita, Daniel Faria, André O. Falcão, Phillip Lord, Francisco M. Couto

**Affiliations:** 1 LaSIGE, Faculty of Sciences, University of Lisboa, Lisboa, Portugal; 2 School of Computing Science, Newcastle University, Newcastle-upon-Tyne, United Kingdom; University of California San Diego, United States of America

## Abstract

In recent years, ontologies have become a mainstream topic in biomedical research. When biological entities are described using a common schema, such as an ontology, they can be compared by means of their annotations. This type of comparison is called semantic similarity, since it assesses the degree of relatedness between two entities by the similarity in meaning of their annotations. The application of semantic similarity to biomedical ontologies is recent; nevertheless, several studies have been published in the last few years describing and evaluating diverse approaches. Semantic similarity has become a valuable tool for validating the results drawn from biomedical studies such as gene clustering, gene expression data analysis, prediction and validation of molecular interactions, and disease gene prioritization.

We review semantic similarity measures applied to biomedical ontologies and propose their classification according to the strategies they employ: node-based versus edge-based and pairwise versus groupwise. We also present comparative assessment studies and discuss the implications of their results. We survey the existing implementations of semantic similarity measures, and we describe examples of applications to biomedical research. This will clarify how biomedical researchers can benefit from semantic similarity measures and help them choose the approach most suitable for their studies.

Biomedical ontologies are evolving toward increased coverage, formality, and integration, and their use for annotation is increasingly becoming a focus of both effort by biomedical experts and application of automated annotation procedures to create corpora of higher quality and completeness than are currently available. Given that semantic similarity measures are directly dependent on these evolutions, we can expect to see them gaining more relevance and even becoming as essential as sequence similarity is today in biomedical research.

## Introduction

Comparison and classification have been central pillars of biology since Linnaeus proposed his taxonomy and Darwin observed the mockingbirds on the Galapagos Islands. Like most scientific knowledge, biological laws and models are derived from comparing entities (such as genes, cells, organisms, populations, species) and finding their similarities and differences. However, biology is unlike other sciences in that its knowledge can seldom be reduced to mathematical form. Thus, biologists either record their knowledge in natural language—for example, in scientific publications—or they must seek other forms of representation to organize it, such as classification schemes. When new entities arise, biologists approach them by comparing them to known entities and making inferences according to their degree of similarity.

Comparing entities is not always trivial. For instance, while the sequences or structures of two gene products can be compared directly (through alignment algorithms), the same is not true of their functional aspects. The difference is that sequences and structures have an objective representation and measurable properties, whereas functional aspects have neither. This does not mean that it is impossible to compare functional aspects, but that to be compared they must be expressed in a common and objective form.

The advent of automated sequencing has had deep repercussions on knowledge representation in biology. As experimental methods shift in scope from the gene level to the genomic level, computational analysis is proving essential in handling the increasing amount of data. Thus it has become crucial to adopt common and objective knowledge representations to help knowledge sharing and computer reasoning. This need led to the development of ontologies for goals such as annotating gene products (Gene Ontology), annotating sequences (Sequence Ontology), and annotating experimental assays (Microarray and Gene Expression Data Ontology).

The adoption of ontologies for annotation provides a means to compare entities on aspects that would otherwise not be comparable. For instance, if two gene products are annotated within the same schema, we can compare them by comparing the terms with which they are annotated. While this comparison is often done implicitly (for instance, by finding the common terms in a set of interacting gene products), it is possible to do an explicit comparison with semantic similarity measures. Within the context of this article, we define a semantic similarity measure as a function that, given two ontology terms or two sets of terms annotating two entities, returns a numerical value reflecting the closeness in meaning between them.

The Gene Ontology (GO) [1] is the main focus of investigation of semantic similarity in molecular biology, not only because it is the ontology most widely adopted by the life sciences community, but also because comparing gene products at the functional level is crucial for a variety of applications. Semantic similarity applied to the GO annotations of gene products provides a measure of their functional similarity. From this point forward, we will use the term “functional similarity” when referring to the similarity between two gene products given by the semantic similarity between the sets of GO terms with which they are annotated. As such, the semantic similarity measures and the studies reviewed in this article are presented in the context of GO, notwithstanding the fact that they are applicable to other biological ontologies.

**Figure 1:**
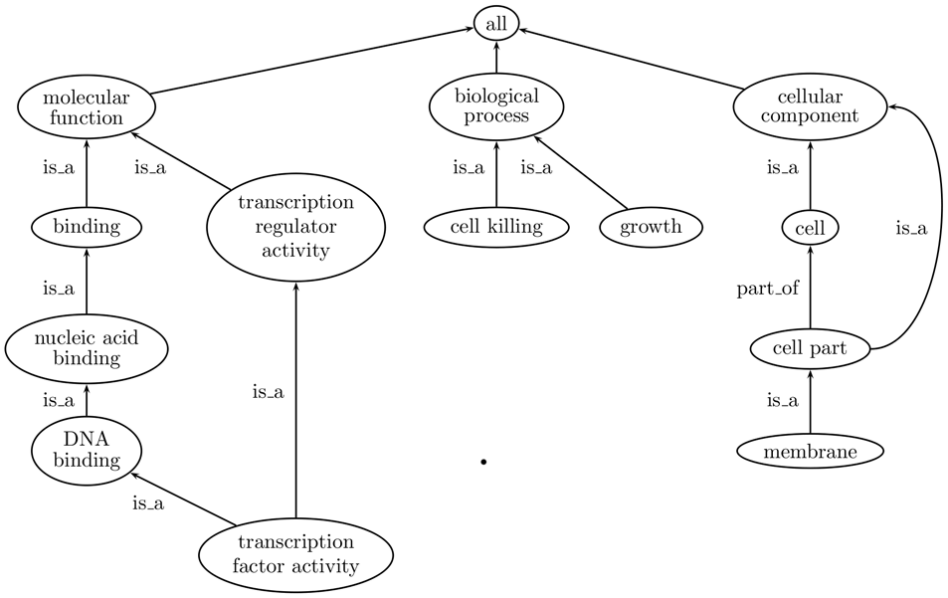
Section of the GO graph showing the three aspects (*molecular function*, *biological process*, and *cellular component*) and some of their descendant terms. The fact that GO is a DAG rather than a tree is illustrated by the term “transcription factor activity” which has two parents. An example of a *part of* relationship is also shown between the terms cell part and cell.

GO provides a schema for representing gene product function in the cellular context. Figure 1 shows how GO is structured as three independent directed acyclic graphs (DAGs) that correspond to orthogonal categories of gene product function: *molecular function*, *biological process*, and *cellular component*. The nodes in the graph represent terms that describe components of gene product function. GO links the terms to each other by relationships, most commonly of the types ‘*is a*’ and ‘*part of*’, the former expressing a simple class–subclass relationship and the latter expressing a part–whole relationship. Gene products that are described by GO terms are said to be annotated with them, either directly or through inheritance, since annotation to a given term implies annotation to all of its ancestors (*true path rule*). The Gene Ontology Consortium is responsible for developing and maintaining GO terms, their relationships, and their annotations to genes and gene products of the collaborating databases. Moreover, GO Consortium is also responsible for developing tools that support the creation, maintenance, and use of all this information.

## Classification of Semantic Similarity Measures

Several approaches are available to quantify semantic similarity between terms or annotated entities in an ontology represented as a DAG such as GO. This article distinguishes these approaches in the following way:

**Scope:** Which entities they intend to compare, that is, GO terms versus gene products;
**Data source:** Which components of the ontology they use, i.e., edges versus nodes;
**Metric:** How they quantify and combine the information stored on those components.

### Comparing Terms

**Figure 2:**
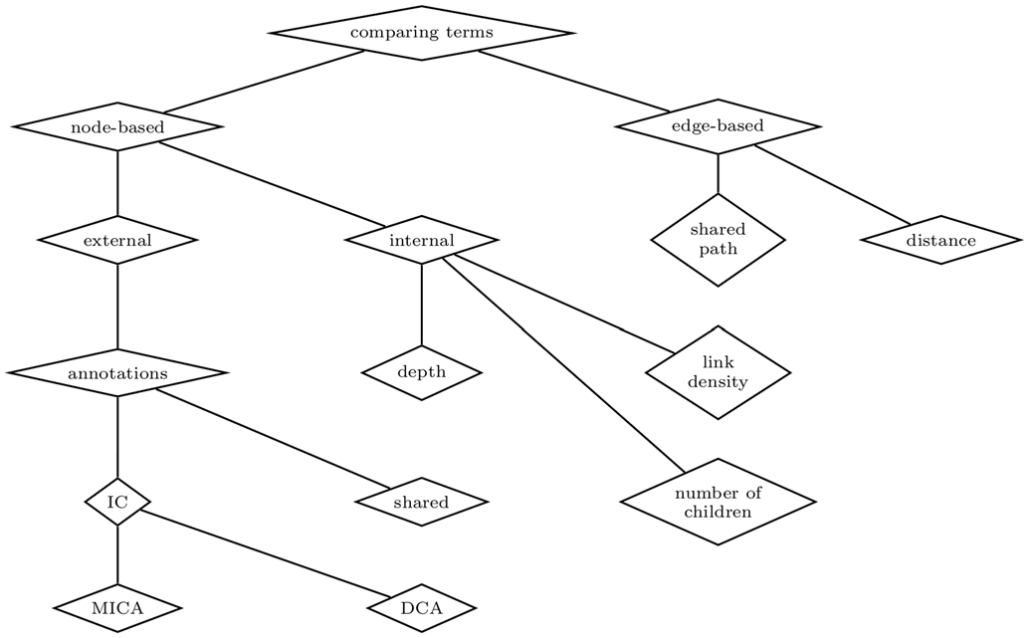
Main approaches for comparing terms: *node-based* and *edge-based* and the techniques used by each approach. DCA, disjoint common ancestors; IC, information content; MICA, most informative common ancestor.

There are essentially two types of approaches for comparing terms in a graph-structured ontology such as GO: edge-based, which use the edges and their types as the data source; and node-based, in which the main data sources are the nodes and their properties. We summarize the different techniques employed in these approaches in Figure 2 and describe them in the following sections. Note that there are other approaches for comparing terms that do not use semantic similarity—for example, systems that select a group of terms that best summarize or classify a given subject based on the discrete mathematics of finite partially ordered sets [2].

#### Edge-based

Edge-based approaches are based mainly on counting the number of edges in the graph path between two terms [3]. The most common technique, *distance*, selects either the shortest path or the average of all paths, when more than one path exists. This technique yields a measure of the distance between two terms, which can be easily converted into a similarity measure. Alternatively, the *common path* technique calculates the similarity directly by the length of the path from the lowest common ancestor of the two terms to the root node [4].

While these approaches are intuitive, they are based on two assumptions that are seldom true in biological ontologies: (1) nodes and edges are uniformly distributed [5], and (2) edges at the same level in the ontology correspond to the same semantic distance between terms. Several strategies have been proposed to attenuate these issues, such as weighting edges differently according to their hierarchical depth, or using node density and type of link [6]. However, terms at the same depth do not necessarily have the same specificity, and edges at the same level do not necessarily represent the same semantic distance, so the issues caused by the aforementioned assumptions are not solved by those strategies.

#### Node-based

Node-based approaches rely on comparing the properties of the terms involved, which can be related to the terms themselves, their ancestors, or their descendants. One concept commonly used in these approaches is information content (IC), which gives a measure how specific and informative a term is. The IC of a term *c* can be quantified as the negative log likelihood,
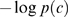
where *p*(*c*) is the probability of occurrence of *c* in a specific corpus (such as the UniProt Knowledgebase), being normally estimated by its frequency of annotation. Alternatively, the IC can also be calculated from the number of children a term has in the GO structure [7], although this approach is less commonly used.

The concept of IC can be applied to the common ancestors two terms have, to quantify the information they share and thus measure their semantic similarity. There are two main approaches for doing this: the most informative common ancestor (MICA technique), in which only the common ancestor with the highest IC is considered [8]; and the disjoint common ancestors (DCA technique), in which all disjoint common ancestors (the common ancestors that do not subsume any other common ancestor) are considered [9].

Approaches based on IC are less sensitive to the issues of variable semantic distance and variable node density than edge-based measures [8], because the IC gives a measure of a term's specificity that is independent of its depth in the ontology (the IC of a term is dependent on its children but not on its parents). However, the IC is biased by current trends in biomedical research, because terms related to areas of scientific interest are expected to be more frequently annotated than other terms. Nevertheless, the use of the IC still makes sense from a probabilistic point of view: it is more probable (and less meaningful) that two gene products share a commonly used term than an uncommonly used term, regardless of whether that term is common because it is generic or because it is related to a hot research topic.

Other node-based approaches include looking at the number of shared annotations, that is, the number of gene products annotated with both terms [10]; computing the number of shared ancestors across the GO structure; and using other types of information such as node depth and node link density (i.e., node degree) [11].

### Comparing Gene Products

**Figure 3:**
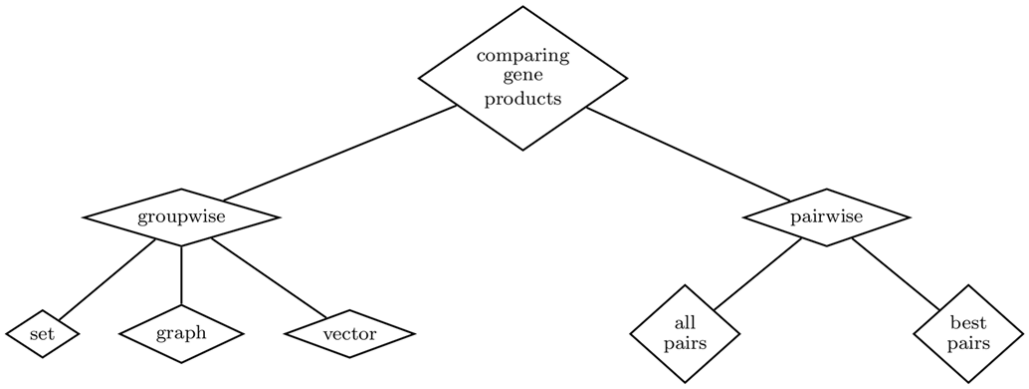
Main approaches for comparing gene products: *pairwise* and *groupwise* and the techniques used by each approach.

Gene products can be annotated with several GO terms within each of the three GO categories. Gene product function is often described by several *molecular function* terms, and gene products often participate in multiple *biological processes* and are located in various *cellular components*. Thus, to assess the functional similarity between gene products (within a particular GO category) it is necessary to compare sets of terms rather than single terms. Several strategies have been proposed for this, which we have divided into two categories: pairwise and groupwise approaches, as shown in Figure 3.

#### Pairwise

Pairwise approaches measure functional similarity between two gene products by combining the semantic similarities between their terms. Each gene product is represented by its set of direct annotations, and semantic similarity is calculated between terms in one set and terms in the other (using one of the approaches described previously for comparing terms). Some approaches consider every pairwise combination of terms from the two sets (*all pairs* technique), while others consider only the best-matching pair for each term (*best pairs* technique). A global functional similarity score between the gene products is obtained by combining these pairwise semantic similarities, with the most common combination approaches being the average, the maximum, and the sum.

#### Groupwise

Groupwise approaches do not rely on combining similarities between individual terms to calculate gene product similarity, but calculate it directly by one of three approaches: set, graph, or vector.

In set approaches only direct annotations are considered and functional similarity is calculated using set similarity techniques.

In graph approaches gene products are represented as the subgraphs of GO corresponding to all their annotations (direct and inherited). Functional similarity can be calculated either using graph matching techniques or, because these are computationally intensive, by considering the subgraphs as sets of terms and applying set similarity techniques.

In vector approaches a gene product is represented in vector space, with each term corresponding to a dimension, and functional similarity is calculated using vector similarity measures. Vectors can be binary, with each dimension denoting presence or absence of the term in the set of annotations of a given gene product, or scalar, with each dimension representing a given property of the term (for example, its IC).

## Survey of Semantic Similarity Measures

Since the first application of semantic similarity in biology, by Lord et al. [12], several semantic similarity measures have been developed for use with GO, as shown in Table 1. The following sections present a survey of semantic similarity measures proposed within the context of GO.

**Table 1:** Summary of term measures, their approaches, and their techniques.

Measure	Approach	Techniques
Resnik [8]	Node-based	MICA
Lin [13]	Node-based	MICA
Jiang and Conrath [14]	Node-based	MICA
GraSM [9]	Node-based	DCA
Schlicker et al. [16]	Node-based	MICA
Wu et al. [22]	Edge-based	Shared path
Wu et al. [23]	Edge-based	Shared path; distance
Bodenreider et al. [17]	Node-based	Shared annotations
Othman et al. [11]	Hybrid	IC/depth/number of children; distance
Wang et al. [25]	Hybrid	Shared ancestors
Riensche et al. [18]	Node-based	IC/MICA; shared annotations
Yu et al. [20]	Edge-based	Shared path
Cheng et al. [21]	Edge-based	Shared path
Pozo et al. [24]	Edge-based	Shared path

### Measures for Terms

#### Node-based

The most common semantic similarity measures used with GO have been Resnik's, Lin's, and Jiang and Conrath's, which are node-based measures relying on IC [8],[13],[14]. They were originally developed for the WordNet, and then applied to GO [12],[15]. Resnik measures similarity between two terms as simply the IC of their most informative common ancestor (MICA):
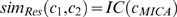
(1)


While this measure is effective in determining the information shared by two terms, it does not consider how distant the terms are from their common ancestor. To take that distance into account, Lin's and Jiang and Conrath's measures relate the IC of the MICA to the IC of the terms being compared:
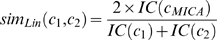
(2)

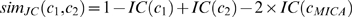
(3)


However, being relative measures, 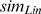 and 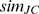 are displaced from the graph. This means that these measures are proportional to the IC differences between the terms and their common ancestor, independently of the absolute IC of the ancestor.

To overcome this limitation, Schlicker et al. [16] have proposed the relevance similarity measure, which is based on Lin's measure, but uses the probability of annotation of the MICA as a weighting factor to provide graph placement.
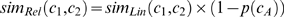
(4)


A constraint all of these measures share is that they look only at a single common ancestor (the MICA) despite the fact that GO terms can have several DCA. To avoid this, Couto et al. [9] proposed the GraSM approach, which can be applied to any of the measures previously described, and where the IC of the MICA is replaced by the average IC of all DCA.

Bodenreider et al. [17] developed a node-based measure that also uses annotation data but does not rely on information theory. It represents each GO term as a vector of all gene products annotated with it, and measures similarity between two terms by computing the scalar product of their vectors.

Riensche et al. used coannotation data to map terms between different GO categories and calculate a weighting factor, which can then be applied to a standard node-based semantic similarity measure [18].

#### Edge-based

Within the edge-based approaches, Pekar and Staab proposed a measure based on the length of the longest path between two terms' lowest common ancestor and the root (maximum common ancestor depth), and on the length of the longest path between each of the terms and that common ancestor [19]. It is given by the expression
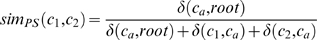
(5)where *δ*(*c*_1_,*c*_2_) is the length in number of edges of the longest distance between term *c*_1_ and term *c*_2_. This measure was first applied to GO by Yu et al. [20].

Cheng et al. also proposed a maximum common ancestor depth measure, but weighted each edge to reflect depth [21]. Wu et al. proposed a nonweighted maximum common ancestor depth measure [22]. An adjustment of this measure was proposed by Wu et al., introducing the distance to the nearest leaf node and the distance to the lowest common ancestor to take term specificity into account [23].

A distinct approach was proposed by Pozo et al. [24], where a “Functional Tree” for *molecular function* terms is first derived from their co-occurrence in the same set of Interpro entries, and semantic similarity between two terms is calculated from the height of their lowest common ancestor in this “Functional Tree” rather than in the GO graph. With this approach, the authors intend to reveal natural biological links between the terms.

#### Hybrid

Wang et al. [25] developed a hybrid measure in which each edge is given a weight according to the type of relationship. For a given term *c*_1_ and its ancestor *c_a_*, the authors define the semantic contribution of *c_a_* to *c*_1_, as the product of all edge weights in the “best” path from *c_a_* to *c*_1_, where the “best” path is the one that maximizes the product. Semantic similarity between two terms is then calculated by summing the semantic contributions of all common ancestors to each of the terms and dividing by the total semantic contribution of each term's ancestors to that term. Othman et al. proposed a hybrid distance measure in which each edge is weighted by node depth, node link density, and difference in IC between the nodes linked by that edge [11].

### Measures for Gene Products

Several measures for calculating the functional similarity between gene products have also been developed, as shown in Tables 2 and 3.

**Table 2:** Summary of pairwise approaches.

Measure	Approach	Techniques	Term Comparison
Lord et al. [12]	All pairs	Average	Resnik/Lin/Jiang
Sevillla et al. [26]	All pairs	Maximum	Resnik/Lin/Jiang
Riensche et al. [18] (XOA)	All pairs	Maximum	XOA
Azuaje et al. [27]	Best pairs	Average	Resnik/Lin/Jiang
Couto et al. [9]	Best pairs	Average	GraSM+(Resnik/Lin/Jiang)
Schlicker et al. [16] (funSim)	Best pairs	Average	simRel
Wang et al. [25]	Best pairs	Average	Wang
Tao et al. [61] (ITSS)	Best pairs	Average Min. threshold	Lin
Pozo et al. [24]	Best pairs	Average	Pozo
Lei et al. [29]	All pairs Best pairs^a^	Average Max, Sum	Depth of LCA

a Lei et. al also consider exact matches only.

**Table 3:** Summary of groupwise approaches.

Measure	Approach	Techniques	Weighting
Lee et al. [30] (TO)	Graph-based	Term overlap	None
Mistry et al. [31] (NTO)	Graph-based	Term overlap, Normalized	None
Gentleman [33] (simLP)	Graph-based	Shared-path	None
Gentleman [33] (simUI)	Graph-based	Jaccard	None
Martin et al. [32] (GOToolBox)	Graph-based	Czekanowski-Dice, Jaccard	None
Pesquita et al. [44] (simUI)	Graph-based	Jaccard	IC
Ye et al. [35]	Graph-based	LCA, Normalized	None
Cho et al. [36]	Graph-based	LCA	IC
Lin et al. [37]	Graph-based	Intersection	Annotation set probability
Yu et al. [38]	Graph-based	LCA	Annotation set probability
Sheehan et al. [39] (SSA)	Graph-based	Resnik, Lin	Annotation set probability
Huang et al. [40]	Vector-based	Kappa-statistic	None
Chabalier et al. [41]	Vector-based	Cosine	IC

#### Pairwise

The most common methods of measuring gene product functional similarity have been pairwise approaches based on node-based term measures, namely, Resnik's, Lin's, and Jiang and Conrath's. Lord et al. were the first to apply these measures, using the average of all pairwise similarities as the combination strategy [12]; Sevilla et al. applied them using the maximum of the pairwise similarities instead [26]; while Couto et al. and Azuaje et al. opted for a composite average in which only the best-matching term pairs are considered (best-match average) [9],[27]. Schlicker et al. all proposed a variation of the best-match average, by combining semantic similarities for both *molecular function* and *biological process* aspects of GO [16]; while Tao et al. used a threshold of minimum similarity for selecting best-matching term pairs, and considered only reciprocal pairs to reduce the noise [28]. Riensche et al. also employed the maximum combination strategy, but introduced a variation to allow comparison of terms from different aspects of GO (see node-based term measures) [18].

Pairwise approaches have also been applied to edge-based measures: Wang et al. and Pozo et al. used a best-match average combination strategy with their measures [24],[25], and Lei et al. tested a number of different combination approaches, including the average, maximum, and sum for all pairs, best pairs, and only exact matches [29].

Of the several combination strategies employed in pairwise measures, the best-match average variants are the best overall. The maximum approach can answer the question of whether two gene products share a functional aspect, but is unsuitable to assess their global similarity, as it is indifferent to the number of functional aspects they share and to the number of functional aspects in which they differ. For instance, a gene product *A* with terms *t1* and *t2* will be considered 100% similar to a gene product *B* with terms *t1* and *t3* under the maximum approach, which obviously does not reflect the differences between the gene products. As for the average approach, because it makes an all-against-all comparison of the terms of two gene products, it will produce counterintuitive results for gene products that have several distinct functional aspects. For instance, two gene products, *A* and *B*, that share the same two unrelated terms, *t1* and *t2*, will be 50% similar under the average approach, because similarity will be calculated between both the matching and the opposite terms of the two gene products. The best-match average approach provides a good balance between the maximum and average approaches by considering all terms but only the most significant matching.

#### Groupwise

Purely set-based approaches are not common, because few measures consider only direct annotations, but many graph-based approaches use set similarity techniques to simplify the problem of graph matching. The first graph-based measure to be applied to GO was that of Lee et al. [30], in which the similarity between gene products is defined by the number of terms they share (term overlap [TO]). More recently, Mistry et al. [31] proposed a normalized version of Lee's measure (NTO), in which the number of overlapping terms is divided by the annotation set size for the gene with the lower number of annotations. GOToolBox also implements some set similarity techniques applied to GO graphs, namely Czekanowski-Dice and Jaccard [32]. Gentleman's simLP and simUI measures were also among the first graph-based measures to be applied to GO [33]: simLP extends the maximum common ancestor depth concept to gene products, so two gene products are as similar as the depth of the term that is the lowest common ancestor to all the terms' direct annotations; whereas simUI considers gene products as the set of terms in their annotation subgraphs, and uses the Jaccard index to calculate the similarity between them:
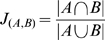
(6)


Based also on the Jaccard index, Pesquita et al. have proposed the simGIC measure, in which each GO term is weighted by its IC [34].

Ye et al. proposed a normalized version of simLP that takes into account the minimum and maximum depths within each GO category [35].

Cho et al. developed a simpler groupwise approach, in which the semantic similarity between two gene products is given by the information content of the most informative term they share [36]. This produces the same result as Resnik's measure with the maximum combination strategy, but is simpler to apply to gene products, as it does not require computing pairwise term similarities.

Other graph-based measures consider the probability of a gene product being annotated with a particular set of terms (annotation set probability). Lin et al. calculate the similarity between two gene product subgraphs as the frequency of occurrence of the graph resulting from the intersection of both subgraphs, that is, the frequency of gene products whose annotation subgraph contains the intersect graph [37]. Yu et al. proposed the “total ancestry similarity” measure, a probabilistic approach in which similarity is given by the probability that any two gene products have exactly the same set of lowest common ancestors as the two gene products being compared [38]. The SSA algorithm by Sheehan et al. is based on the probability of any given gene product being annotated with the nearest common ancestors of two gene products. This probability is then transformed into an IC measure that the authors use to compute Resnik's and Lin's measures to obtain a final gene product similarity value [39]. This algorithm also considers the types of relations between the terms in the subgraphs and corrects the number of annotations for the parent term in a *part_of* relation, if its number of annotations is smaller than its child's, to comply with the logic that if the part exists, necessarily the whole does too.

As for vector-based approaches, Huang et al. developed a gene product similarity measure based on annotation profiles that includes GO terms as well as many other derived from varied sources for the tool DAVID [40]. Each gene product is represented as a binary vector with each term having the value 1 if it is annotated to the gene product or 0 otherwise. Similarity is calculated through kappa-statistics, which are a co-occurrence probability measure corrected for chance.

Chabalier et al. also consider gene products as a vector of all GO terms, but weight them according to their IC. Semantic similarity is then calculated through the cosine similarity algorithm, which is commonly used to measure document similarity in information retrieval [41]:
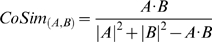
(7)


## Evaluation of Semantic Similarity Measures

Given the variety of approaches and measures for semantic similarity, a fundamental question arises: How well does each measure capture the similarity in function between two gene products?

Addressing this question is not trivial, because there is no direct way to ascertain the true functional similarity between two gene products. If there were, there would be no need to apply semantic similarity in the first place. However, there are independent properties, such as sequence similarity or coexpression data, that can be used as measures of similarity at different levels, and by correlating semantic similarity with such properties it is feasible to assess how well a given measure captures the similarity between gene products.

The choice of how to evaluate measures is still a subject of debate, because no gold standard and few global comparative studies exist. Furthermore, most authors test only a few measures of interest for their particular applications. Table 4 summarizes the most prominent studies and the best measure identified by them.

**Table 4:** Summary of assessment studies performed on semantic similarity measures in GO, detailing the properties used in the evaluation and the best performing measures.

Study	Standard	Best Measure
Lord et al. [15]	Sequence similarity	Resnik (average)
Wang et al. [42]	Gene expression	None
Sevillla et al. [26]	Gene expression	Resnik (max)
Couto et al. [9]	Family similarity	Jiang and Conrath
Schlicker et al. [16]	Sequence and family similarity	Schlicker et al.
Lei et al. [29]	Subnuclear location	TO
Guo et al. [43]	Human regulatory pathways	Resnik (average)
Wang et al. [25]	Clustering	Wang et al.
Pesquita et al. [44]	Sequence similarity	simGIC
Xu et al. [45]	PPI/gene expression	Resnik(Max)
Mistry et al. [31]	Sequence similarity	TO/Resnik(Max)

The first assessment study was done by Lord et al., who tested Resnik's, Lin's, and Jiang and Conrath's measures against sequence similarity using the average combination approach [15]. They used linear correlation as the evaluation criterion, concluding that it was highest for the molecular function aspect, as would be expected given the strong relationship between function and sequence. Of the three measures tested, Resnik's measure had consistently the highest correlation.

Later, Wang et al. tested the same measures against gene coexpression data, also using linear correlation. Although a high correlation was found, no conclusion was drawn about the relative performance of the measures [42].

Sevilla et al. also used gene coexpression data, but tested these measures with the maximum combination approach rather than the average. In agreement with Lord et al., they found Resnik's measure to perform best, and found the biological process aspect to have the highest correlation with gene coexpression. This result was far from unexpected, since genes with similar expression patterns are likely to be involved in the same biological processes [26].

Couto et al. tested the GraSM variation of these measures using a best-match average combination strategy, correlating sequence similarity with the number of Pfam families shared. They found the highest correlation with Jiang and Conrath's measure and the biological process aspect [9].

Schlicker et al. compared their measure with Resnik's measure by viewing the distribution of sequence similarity over semantic similarity, considering discrete levels. They found their measure to perform best, particularly in distinguishing orthologous gene products from gene products with other levels of sequence similarity, for both molecular function and biological process aspects [16].

Lei et al. tested several semantic similarity measures in an application for predicting subnuclear location, using the multi-class classification results (Matthews correlation coefficient) as an evaluation criterion [29]. Regarding term measures, these authors compared Resnik's measure with the simLP measure (adapted for term similarity) and an exact term-matching approach, finding them not to be significantly different in performance. They also tested several combination strategies for applying simLP to gene product similarity, concluding that the sum of exact-matching term pair similarities produced the best results. This outcome is not surprising, given the precise nature of their application, as the subnuclear location of a gene product will often be related to the presence of specific GO terms.

Guo et al. evaluated simUI, simLP, Resnik's, Lin's, and Jiang and Conrath's measures on their ability to characterize human regulatory pathways. They concluded that the pairwise approaches tested performed better than groupwise approaches, with Resnik's measure being the best performing overall [43].

Wang et al. tested their measure against Resnik's by clustering gene pairs according to their semantic similarity and comparing the resulting clusters with a human perspective. They showed that their measure produced results closer to human expectations [25].

Recently, Pesquita et al. have done an evaluation of semantic similarity measures by modeling their behavior as function of sequence similarity. These authors compared Resnik's, Lin's, and Jiang and Conrath's measures, using several combination strategies, and also the groupwise measures simUI and simGIC, showing that the relationship between semantic similarity and sequence similarity is not linear for any of these measures. They found that the average and maximum combination strategies produce worse results than the best-match average and, consistent with Lord's and Sevilla's results, found that Resnik's measure performs better than the other two term-based measures. Overall the best measure for gene product similarity was shown to be simGIC, slightly surpassing Resnik's measure using the “pairwise-best pairs-average” approach in resolution [44].

Xu et. al have also conducted an evaluation study using protein–protein interactions and gene expression datasets of *Saccharomyces cerevisiae* as the standard [45]. They tested the maximum and average techniques with Resnik's term similarity, and also Tao's, Schlicker's, and Wang's measures with receiver operator characteristic (ROC) analysis. The maximum approach was found to be the best performer in all GO categories. A positive influence of the number of annotations per gene product was also found.

Mistry et al. evaluated eleven measures: Resnik, Lin, and Jiang and Conrath with both the average and maximum approaches; three vector-based measures (cosine, weighted cosine, and kappa); and TO and NTO. They investigated the correlation between measures and the correlation with sequence similarity. They found a good correlation between TO and Resnik's maximum and average. These three measures also correlated well to sequence similarity, with TO presenting the highest correlation.

What we can draw from these studies is that there is no clear best measure for comparing terms or gene products. Different measures have performed differently under different circumstances, and a given measure can be well suited for a specific task but perform poorly in another. For instance, simUI was found by Guo et al. to be the weakest measure when evaluated for its ability to characterize human regulatory pathways, while Pesquita et al. found it to be fairly good when evaluated against sequence similarity. However, one result has been obtained consistently: pairwise measures using Resnik's term similarity outperform Lin's and Jiang & Conrath's methods in all studies except family similarity.

There is also no clear best strategy for evaluating GO-based semantic similarity measures; there are arguments for and against most of the strategies employed. For instance, sequence similarity is well known to be related to functional similarity, but it is just as well known that there are gene products with similar sequences but distinct functions and vice-versa. Another example are Pfam families, which are related to global functional aspects of gene products, but will likely not be suitable to compare with detailed GO annotations.

## Semantic Similarity Implementations

The rise in number of semantic similarity measures was accompanied by the development of tools to calculate them. Currently available tools fall into three categories: Web tools, standalone tools, or R packages (see Table 5).

**Table 5:** Tools for GO-based semantic similarity measures.

Tool	Format Available	Measures Implemented	Input Size^a^	Annotation Types	Extras
FuSSiMeG	Web	Several	2	All	None
GOToolBox	Web	Several	Unlimited	Single	Representation, Clustering, Semantic retrieval
ProteInOn	Web	Several	10	All/manual	Protein interaction
G-SESAME	Web	Wang et al. [25]	2	All manual, Single manual	Clustering, Filter by species
FunSimMat	Web	Several	Unlimited	All	Filter by protein family, Filter by species
DynGO	Standalone	AVG(Resnik)	Unlimited	All ECs	Visualization, Browsing, Semantic retrieval
UTMGO	Standalone	Othman et al. [11]	NA	*IEA*/non-*IEA*	Semantic retrieval of terms
SemSim	R	Several	NA	all/non-*IEA*	Support for clustering, filter by species
GOvis	R	simLP+simUI	NA	All	Visualization
csbl.go	R	Several	NA	NA	Clustering

a Acceptable number of terms or gene products.

Web tools, such as FuSSiMeG (http://xldb.fc.ul.pt/rebil/ssm) [46], GOToolBox (http://burgundy.cmmt.ubc.ca/GOToolBox) [32], ProteInOn (http://xldb.fc.ul.pt/biotools/proteinon) [47], FunSimMat (http://funsimmat.bioinf.mpi-inf.mpg.de) [48], and G-SESAME (http://bioinformatics.clemson.edu/G-SESAME) [25], are readily available and simple to use, and their maintenance and updating are at the expense of the provider. However, they are limited to a pre-set number of options.

Standalone applications, such as DynGO (http://gauss.dbb.georgetown.edu/liblab/dyngo.html) [49] and UTMGO (available upon request) [11], have the advantages that they are not limited in the size of input data (unlike many Web tools) and can support heavier computations (DynGO supports semantic retrieval of both similar terms and gene products, while UTMGO supports retrieval of similar terms). However, they require a local installation, which can be less appealing for some users (DynGO in particular works as a server–client application), and must be updated by the end user.

The R packages SemSim (http://www.bioconductor.org/packages/2.2/bioc/html/SemSim.html) and GOvis (http://bioconductor.org/packages/2.3/bioc/html/GOstats.html) are part of the Bioconductor project, which comprises many R packages for bioinformatics and biostatistics applications. A more recent R based package for semantic similarity, csbl.go (http://csbi.ltdk.helsinki.fi/csbl.go/) [50], also implements semantic similarity-based clustering, but it relies on users to load annotation data. The main advantage of these implementations is the possibility of integration between the semantic similarity and other packages, such as visualization tools or statistical analysis.

Although no single tool implements all existing semantic similarity measures, FuSSiMeG, ProteInOn, FunSimMat, SemSim, and csbl.go provide a wide range of them, and enable the user to choose one or even to compare several (in the case of FunSimMat). Most of the tools mentioned above also provide other types of services and information, such as protein interactions (ProteInOn), GO graph visualization (GOvis, DynGO), GO browsing (DynGO), and clustering (GOToolBox, G-SESAME, csbl.go).

## Semantic Similarity Applications

The application of semantic similarity allows gene products to be compared at different levels. This section presents several scenarios in which GO-based semantic similarity measures have been successfully applied.

For instance, GO-based semantic similarity can be used to compare gene products by their biochemical function (*molecular function*), the cellular and supracellular processes in which they are involved (*biological process*), and their cellular or extracellular location (*cellular component*). Comparing the molecular function aspect, we can measure the functional similarity between gene products and gain insight into function-related characteristics such as domains and active sites. The biological process aspect can be related to protein interaction, both physical and indirect (involved in the same process network), and thus can be used to predict them and to analyze coexpression data. The cellular component aspect can be linked to colocalization and in that context be used to validate physical interaction and localization-dependent functions and processes. Overall, GO-based semantic similarity measures have been applied mainly for validating and predicting functions and interactions, and for analyzing transcriptomics and proteomics data.

Automated prediction of gene product function has been a cornerstone of genome annotation, because experimental methods are too costly and time consuming to be able to cope with the size and continuous growth of genetic data [51]. Semantic similarity can be used to assess the performance of automated function prediction methods (as was used in the Automated Function Prediction 2005 Special Interest Group meeting of ISMB 2005 in Detroit, USA) and to validate their results [52],. It has also been used as a component of several function prediction systems, based on protein–protein interactions [54], on structural similarity of protein surface [55], and on clustering using semantic similarity [56]; and to validate automatic annotations [57]. Tao et al. have developed a function prediction system in which annotations are transferred between proteins with the only criterion being their semantic similarity [28].

Semantic similarity can also play an important role in both predicting and validating gene product interactions and interaction networks. Regarding prediction, some authors developed methods based solely on semantic similarity [23],[41], whereas others combined semantic similarity with gene expression data [43],[58],[59]. As for validation of interactions, semantic similarity has been used to select a negative dataset of noninteracting proteins to assess prediction methods [60], to improve the performance of predictions by excluding false positives [61], and to assess the quality of predicted interaction networks by comparing them to experimentally verified interactions [48]. Also in the context of interactions, semantic similarity has been used to extract functional modules from interaction networks [62], to align biological pathways [63] to generate functionally meaningful network subsets [64], and to characterize protein interaction networks to support breast cancer outcome prediction [65].

In the analysis of transcriptomics and proteomics data, the main role of semantic similarity has been to improve the clustering of coexpressed gene products by taking into account their functional similarity [56], [66]–[69]. However, it can also be used to link and compare results from different assays [30], to improve data quality [70], and to validate gene selection for biomedical purposes [71].

Other biological applications of semantic similarity include determining interfold similarity based on sequence/structure fragments [72], evaluating the biological significance of coexpressed chromosome domains [73], predicting domain distances [2], and predicting cellular localization [29]. There are also other applications such as integration of semantic search [75],[76].

Unfortunately, most application studies use only one measure and results are not comparable across studies, making it difficult to assess which measure is best for which purpose. However, based on the few comparative studies that exist, we can identify the most successful measures so far in the three main applications of GO-based semantic similarity: function prediction/validation, protein–protein interaction prediction/validation, and cellular location prediction (see Table 6).

**Table 6:** Best measures for the main applications of GO-based semantic similarity measures.

Application	Best Measure	Reference
Function^a^ p/v	BMA(Resnik)/simGIC	[44]
Protein-protein interaction p/v	Max(Resnik)	[43],[45]
Cellular location prediction	SUM(EM)	[29]

a Identified by sequence similarity.

p/v, prediction/validation.

## Discussion

Semantic similarity measures are dependent on the quality and completeness of both the ontology and the annotated corpus they use. Biomedical ontologies have several irregularities, such as variable edge length (edges at the same level convey different semantic distances), variable depth (terms at the same level have different levels of detail), and variable node density (some areas of the ontology have a greater density of terms than others). These are due to the irregular nature of biomedical knowledge and our limited understanding of it, and should be taken into account by semantic similarity measures, particularly edge-based measures, which are sensible to these issues. There are also problems with the use of annotated corpora in node-based measures, because these are biased by biomedical research interests (since terms related to subjects of research focus are expected to be more frequently annotated than other terms) and limited by current experimental techniques (if the currently most popular technique can discover gene product functional aspects only to a certain level of detail, then more detailed terms will seldom be used for annotation). Despite these issues, the use of IC on semantic similarity measures still makes sense probabilistically, if not always semantically, because commonly used terms are more likely to be shared by gene products than uncommonly used terms.

Another issue particular to GO is that not all annotations are equal, because evidence codes show the type of evidence that supports the annotation. While most evidence codes symbolize experimental methods and the corresponding annotations are manually curated, the most prevalent evidence code (*IEA*) indicates the annotation was inferred electronically (by sequence similarity, from other databases, or from keyword mapping files) and not manually curated. Whether *IEA* annotations should be used or disregarded for semantic similarity is still a subject of open debate, because using them entails a loss of precision but not using them entails a loss of coverage (over 97% of all annotations in the GOA-UniProt database are *IEA*, and less than 2% of the GO terms have non-*IEA* annotations [77]). As the proportion of *IEA* annotations continues to increase, and they steadily improve in quality (up to 91%–100% precision having been reported [78]), there will be fewer reasons to ignore them, and they will eventually be widely adopted. Since *IEA* annotations are usually made by inference through similarity to sequences of model organisms, improvements in the experimental annotation of model organisms will result in higher-quality *IEA* annotations. Meanwhile, perhaps the best way to decide whether to include or discard *IEA* annotations for a particular study is to first analyze the corpus in question and verify if the gene products of interest are well characterized with manually curated annotations or if *IEA* annotations are essential for their characterization. Note that using different sets of annotations (such as all, or just the manually curated ones, etc.) will have an impact in the IC values calculated for the terms, preventing their comparison. It is also important to stress that only results obtained with the same versions of GO's ontology and annotation data are comparable, since changes to both the ontologies and the annotations made with them affects semantic similarity .

An important issue in the evaluation of semantic similarity measures based on the IC is that of data circularity between the data used to evaluate the measures and the GO annotations. For instance, if a given measure is evaluated by correlation with sequence similarity, then using annotations based on sequence similarity (those with evidence codes *ISS* and *IEA*) to calculate the IC leads to data circularity, as there is direct dependency between both data sources. The same is true for the use of annotations inferred by physical interaction (*IPI*) when a measure is evaluated by correlation with protein-protein interactions, and other similar cases. To minimize the effect of data circularity, evaluation studies should (and usually do) remove annotations based on evidence of the same nature as the data used to evaluate the measures.

Data circularity is not the only problem in evaluating GO-based semantic similarity measures. The lack of a gold standard for evaluating semantic similarity makes it hard to assess the quality of the different measures and to find out which are best for which goals. One of the reasons for the continued lack of a gold standard is that measures are often developed for specific goals and evaluated only in that context. Furthermore, there are pros and cons to all data sources used to evaluate semantic similarity. The best solution is likely that a gold standard be designed by experts to cover most applications of semantic similarity, not based on proxies for true functional similarity, such as sequence similarity or gene coexpression.

### How to Choose a Semantic Similarity Measure

Researchers who wish to employ semantic similarity in their work need to spend some time defining their requirements to choose an adequate measure. Since different measures interpret ontology and annotation information in different ways, researchers need to understand the differences and decide which interpretation is best suited to their needs. Below, we outline some of the steps that should be taken before choosing a semantic similarity measure.

1. Identify your scope: Comparing one aspect versus comparing multiple aspects of gene products;
2. Identify your level of detail: Comparing gene products in specific functions or overall similarity; and
3. Analyze the annotations of your dataset: Determining the number of annotations per gene product, including and excluding *IEA* annotations and annotation depth.

When wishing to compare single aspects of gene products, researchers should opt for maximum approaches (“pairwise–all pairs–maximum”). These will give a measure of how similar two gene products are at their most similar aspect. For comparing multiple aspects, the best measures are “pairwise–best pairs–average” or groupwise approaches, since they allow for the comparison of several terms. However, depending on the level of detail desired, “pairwise–best pairs–average” or “groupwise–set” should be used for a higher degree of specificity (since only direct annotations are used) and “groupwise–vector” or graph for a more generalized similarity (since all annotations are used). To further minimize the relevance of specificity, unweighted graph or vector measures can be employed, so that high-level GO terms are not considered less relevant.

However, having to analyze the dataset before deciding which measure to use can be cumbersome to researchers who just need a “quick and dirty” semantic similarity calculation. In this case, researchers should resort to one of the several semantic similarity tools available and use their good judgment in analyzing the results. Most semantic similarity measures proposed so far have shown fair if not good results, and for less detailed analyses any one of them can give a good overview of the similarities between gene products.

### Conclusions

Over the last decade, ontologies have become an increasingly important component of biomedical research studies, because they provide the formalism, objectivity, and common terminology necessary for researchers to describe their results in a way that can be easily shared and reused by both humans and computers. One benefit of the use of ontologies is that concepts, and entities annotated with those concepts, can be objectively compared through the use of semantic similarity measures. Although the use of semantic similarity in the biomedical field is recent, there is already a wide variety of measures available that can be classified according to the approach they use.

There are two main semantic similarity approaches for comparing concepts: edge-based, which rely on the structure of the ontology; and node-based, which rely on the terms themselves, using information content to quantify their semantic meaning. Node-based measures are typically more reliable in the biomedical field because most edge-based measures assume that all relationships in an ontology are either equidistant or have a distance as function of the depth, neither of which is true for existing biomedical ontologies.

Because biomedical entities are often annotated with several concepts, semantic similarity measures for comparing entities need to rely on sets of concepts rather than single concepts. There are two main approaches for this comparison: pairwise, in which entities are represented as lists of concepts that are then compared individually; and groupwise, in which the annotation graphs of each entity are compared as a whole.

Several studies have been conducted to assess the performance of different similarity measures, by correlating semantic similarity with biological properties such as sequence similarity, or other classification schemas such as Pfam. Most measures were shown to perform well, but as few comprehensive studies have been conducted, it is difficult to draw any conclusion about which measure is best for any given goal.

Until now, most research efforts in this area developed novel measures or adapted preexisting ones to biomedical ontologies, with most novel measures sporting an increased complexity compared to previous ones. This increased complexity is mainly a result of more recent measures combining several strategies to arrive at a final score. Although the need for improved measures is unquestionable, this trend fails to answer the most pressing community needs: (1) easy application to both small and large datasets, which would be best achieved by developing tools that are at once easy to use and powerful; and (2) elucidation of which measure is better fitted to the researcher's needs, which would imply comparative studies of all existing measures and approaches.

Although important efforts in these two areas have already been made, semantic similarity is still far from reaching the status of other gene product similarities, such as sequence-based ones, in which fast and reliable algorithms coupled with the development of ever-growing databases has made them the cornerstone of present day molecular biology. One important step forward would be the development of a gold standard for gene product function that would allow the effective comparison of semantic similarity measures. Nevertheless, semantic similarity is not restricted to gene products and it can be expected that, as more biomedical ontologies are developed and used, these measures will soon be applied to different scenarios. It is then crucial that bioinformaticians focus on strategies to make semantic similarity a practical, useful, and meaningful approach to biological questions.
